# Human Skin Permeation Studies with PPARγ Agonist to Improve Its Permeability and Efficacy in Inflammatory Processes

**DOI:** 10.3390/ijms18122548

**Published:** 2017-11-28

**Authors:** Marcelle Silva-Abreu, Lupe Carolina Espinoza, María José Rodríguez-Lagunas, María-José Fábrega, Marta Espina, María Luisa García, Ana Cristina Calpena

**Affiliations:** 1 Department of Pharmacy, Pharmaceutical Technology and Physical Chemistry, Faculty of Pharmacy and Food Sciences, University of Barcelona, 08028 Barcelona, Spain; marcellesabreu@gmail.com (M.S.-A.); lcespinoza@utpl.edu.ec (L.C.E.); m.espina@ub.edu (M.E.); rdcm@ub.edu (M.L.G.); 2 Institute of Nanoscience and Nanotechnology (IN2UB), University of Barcelona, 08028 Barcelona, Spain; 3 Departamento de Química y Ciencias Exactas, Universidad Técnica Particular de Loja, Loja 1101608, Ecuador; 4 Department of Biochemistry and Physiology, Faculty of Pharmacy and Food Sciences, University of Barcelona, 08028 Barcelona, Spain; mjrodriguez@ub.edu (M.J.R.-L.); mjfabrega@ub.edu (M.-J.F.); 5 Institut de Recerca en Nutrició i Seguretat Alimentària (INSA), Universitat de Barcelona (UB), 08028 Barcelona, Spain; 6 Institute of Biomedicine, University of Barcelona, 08028 Barcelona, Spain

**Keywords:** skin permeation, PPAR-γ, pioglitazone, limonene, rosacea, inflammation

## Abstract

Rosacea is the most common inflammatory skin disease. It is characterized by erythema, inflammatory papules and pustules, visible blood vessels, and telangiectasia. The current treatment has limitations and unsatisfactory results. Pioglitazone (PGZ) is an agonist of peroxisome proliferator-activated receptors (PPARs), a nuclear receptor that regulates important cellular functions, including inflammatory responses. The purpose of this study was to evaluate the permeation of PGZ with a selection of penetration enhancers and to analyze its effectiveness for treating rosacea. The high-performance liquid chromatography (HPLC) method was validated for the quantitative determination of PGZ. Ex vivo permeation experiments were realized in Franz diffusion cells using human skin, in which PGZ with different penetration enhancers were assayed. The results showed that the limonene was the most effective penetration enhancer that promotes the permeation of PGZ through the skin. The cytotoxicity studies and the Draize test detected cell viability and the absence of skin irritation, respectively. The determination of the skin color using a skin colorimetric probe and the results of histopathological studies confirmed the ability of PGZ-limonene to reduce erythema and vasodilation. This study suggests new pharmacological indications of PGZ and its possible application in the treatment of skin diseases, namely rosacea.

## 1. Introduction

Rosacea is a chronic inflammatory disease of the skin [1,2,3]. The clinical features appear principally in the central region of the face and include the presence of facial erythema, inflammatory papules and pustules, telangiectasia, and edema [4,5,6]. It predominantly affects women and fair-skinned people and can occur at any age but is more frequent in middle-aged individuals [7,8]. The estimated prevalence of rosacea among the population of Europe and United States has a wide range from less than 1% to more than 20%, likely due to differences in the methods used and the populations studied [9,10]. Furthermore, it has been associated with several comorbidities such as depression and anxiety [11,12], dyslipidemia, hypertension, cardiovascular diseases, and metabolic diseases [13,14,15].

The pathogenesis of the disease has not been totally clarified but several factors implicated in the etiology of the disease have been reported, such as genetic predisposition, alterations of the neurovascular system, and dysregulation of the innate and adaptive immune system [16,17]. Studies about the pathophysiological mechanisms of rosacea suggest activation of pattern recognition receptors (PRRs) that identify components from foreign microorganisms and immunostimulatory products [18,19]. An increase in Toll-like receptors-2 (TLR-2) expression, a family of PRRs, has been observed in inflammatory skin diseases such as rosacea. When TLR-2 is stimulated by triggering factors, it induces the release of antimicrobial peptides (cathelicidin LL-37) or proinflammatory cytokines such as IL-8, IL-1β, and TNF-α [18,20]. In addition, the characterization of inflammatory infiltrate in this disease has revealed the activation of T lymphocytes, particularly T-helper 1 (Th1) and T-helper 17 (Th17) cells, as well as the presence of macrophages and mast cells, which mediate the inflammatory reactions and development of the disease [21,22].

Topical treatments like sodium sulfacetamide, azelaic acid, metronidazole, and the alpha-adrenergic agonist are recommended when there are few papules and pustules [23,24]. If the skin lesions are more extensive, systemic medications like tetracyclines are prescribed [25,26]. Despite these pharmacological options, rosacea remains incurable, and thus its treatment focuses mainly on controlling the symptoms [27]. These limitations, coupled with unsatisfactory therapeutic results, demonstrate the need to develop more targeted and efficacious treatments [23,28].

Recent studies have suggested that the peroxisome proliferator-activated receptor-gamma (PPAR-γ), a nuclear receptor that regulates glucose homeostasis and lipid metabolism, has an important role in adaptive immunity by regulating genes expression involved in inflammatory processes [29]. Therefore, it has been proposed that PPAR-γ agonists could act as negative regulators in T cell differentiation and activation to attenuate inflammatory responses of autoimmune diseases [30]. Pioglitazone (PGZ) is a member of the thiazolidinediones, which is useful to treat type 2 diabetes mellitus (DM) [31,32]. Moreover, previous studies have demonstrated that this drug has the capacity to inhibit the signaling pathways involved in inflammatory and immunologic processes [33,34], suggesting that its application could be an effective treatment of inflammatory processes.

Penetration enhancers are used with the aim to improve the transdermal drug delivery [35]. Several chemicals such as sulphoxides, azones, pyrrolidones, alcohols, glycols, surfactants, fatty acids, essential oils, and terpenes have been proposed for their ability to reversibly decrease the barrier resistance, allowing drug penetration into the skin at a greater rate [36,37]. As a result, penetration enhancers represent an alternative to improve the permeability and, consequently, the duration of drug action.

After having taken into consideration the role of PPAR-γ as an important immunomodulator with anti-inflammatory properties, the aim of this study was to evaluate the efficacy of the PGZ solution with a selection of penetration enhancers to improve its permeability for treating rosacea using an in vivo model.

## 2. Results

### 2.1. Validation of the Analytical Method

All the analytical method data can be found in Supplementary materials. The linearity of the method was evaluated by the obtained equation and regression values from the calibration curves determined by least-squares linear regression analysis of the peak-area ratios of the PGZ standards solutions *versus* concentration. Three calibration curves were made in the range of 1.5–110 μg/mL (Table S1). No single calibration standard point was dropped during the validation and the data indicate good linearity of the proposed method. The equation obtained from the average calibration curves and the correlation coefficient value are shown Figure S1. Precision of the method was evaluated at concentrations of 3, 60, and 110 µg/mL for the linearity range. Obtained results are shown in Table S2. Data are expressed as percentage of coefficient of variation (CV) and precision of method. The accuracy of the method was evaluated in small, medium, and large concentrations of the range of linearity studied by comparing the tested concentration with the theoretical concentration. Obtained results are shown in Table S3. Data are expressed as percentage of relative error and accuracy. Robustness examines the effect that operational parameters have on the results and provides an indication of its reliability during normal usage. It was determined by evaluating retention time with tolerance variations in the flow and mobile phase that are shown in Table S4. Specificity was proven by the analysis of blank control of mobile phase (Figure S2), standard of 30 ppm (Figure S3), blank of the skin as control (Figure S4), and sample of skin permeated with PGZ-limonene (Figure S5). Chromatogram did not show interference at the retention time of PGZ. From the lowest concentration standard (1.5 ppm), the detection limit (LOD) and the limit of quantification (LOQ) were determined based on signal-to-noise ratios of 3:1 and 10:1, respectively. Hence, the LOD for PGZ was set at 0.12 ± 0.28 ng/mL and the LOQ at 0.40 ± 0.52 ng/mL.

### 2.2. Permeation Studies in Human Skin

**Figure 1:**
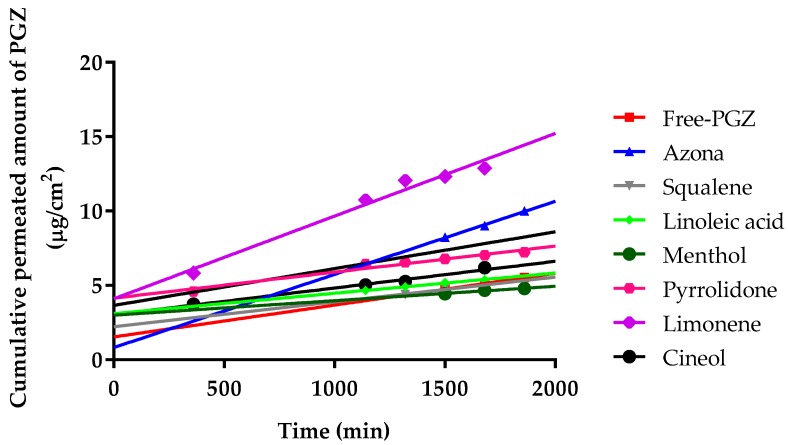
Median cumulative permeated amount of pioglitazone (PGZ) with and without penetration enhancers through human skin, expressed as µg/cm^2^.

The permeation profiles of PGZ with and without penetration enhancers were estimated. The cumulative permeated amount of PGZ (µg) per cm^2^ of human skin in each time interval is shown in Figure 1.

Furthermore, the permeation and prediction parameters of PGZ with permeation enhancers were calculated. The flow (*J_ss_*) and permeability coefficient (*K_p_*) were determined from the cumulative amount of the drug permeated through the skin plotted *versus* time in steady state. Table 1 shows that the limonene presented the highest values for *J_ss_, k_p_, Q_ret_*, and *C_ss_*.

### 2.3. Cytotoxicity Studies and Skin Tolerance Studies

**Figure 2:**
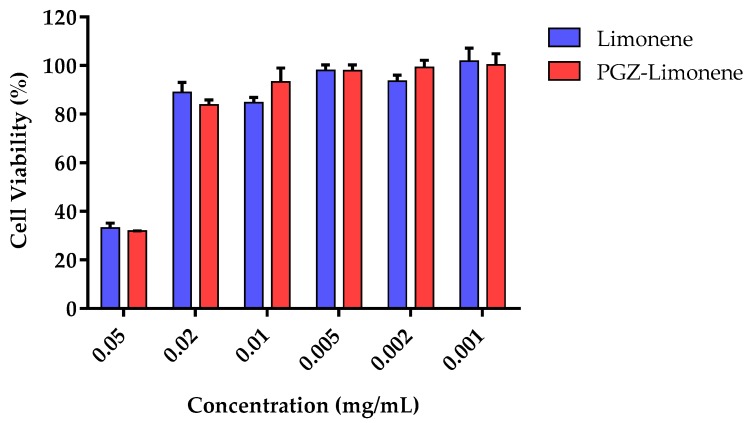
Percentage of cellular viability of immortalized human keratinocytes (HaCaT) cell line exposed to PGZ-limonene and limonene.

The in vitro cytotoxicity assay by 3-(4,5-dimethylthiazol-2-yl)-2,5-diphenyltetrazolium bromide (MTT) was carried out using HaCaT cells after incubation with PGZ-limonene and limonene. Six concentrations were selected according to the level of concentration of each formulation studied. The results showed cell viability greater than 80% in the dilutions assayed from 0.001 to 0.02 mg/mL (Figure 2).

The Draize test was performed in order to evaluate the skin irritation potential of PGZ-limonene and limonene. It had a duration of 72 h using a concentration of 1 mg/mL of PGZ and 5% of limonene. The resulting primary irritation index value of the tested groups were: Control (0.9% NaCl): 0; PGZ-limonene: 0.32 and limonene: 0.43, thereby indicating that PGZ-limonene and limonene are non-irritant.

### 2.4. Efficacy Studies

#### 2.4.1. Colorimetric Parameters

**Figure 3:**
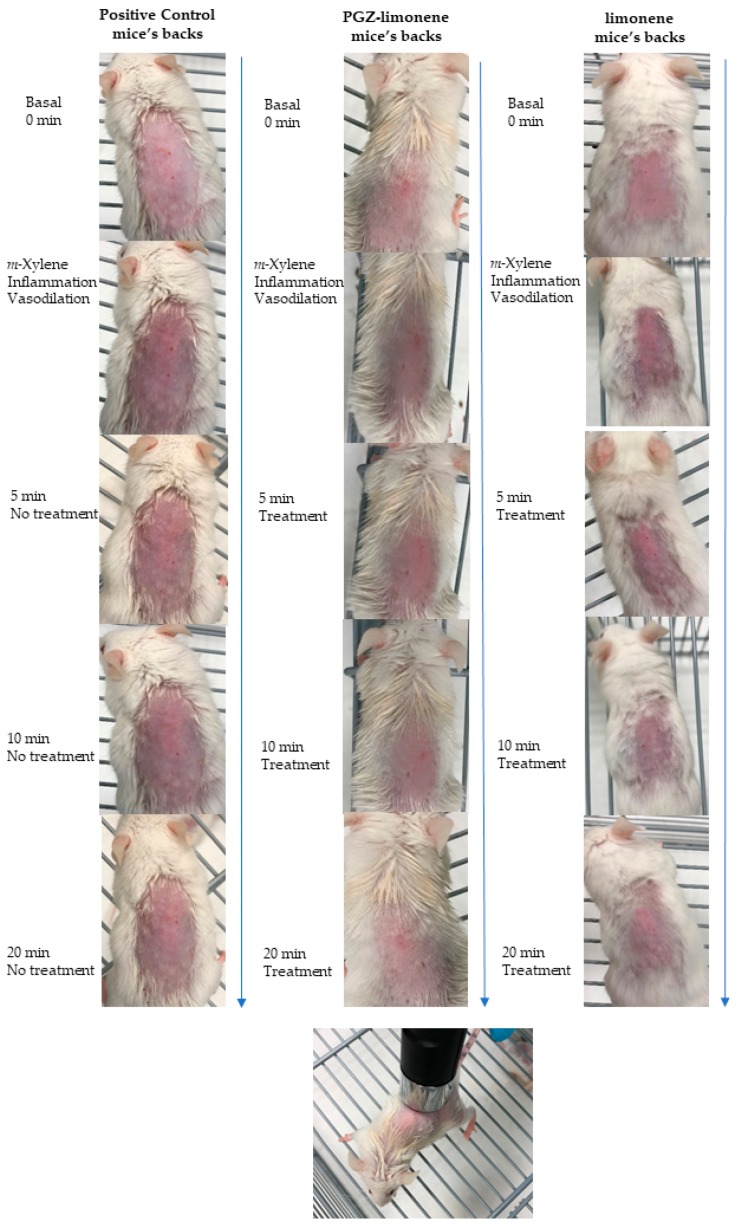
Evolution of erythema shown as skin color sequence on mice’s backs, using PGZ-limonene and limonene compared with positive control. Colors are reproduced from the average values of three basic light components, red, green, and blue (RGB) codes using a Multi Probe Adapter (MPA) 5 Multi Probe adapter from Courage + Khazaka electronic GmbH (Cologne, Germany), equipped with a CL400.

**Figure 4:**
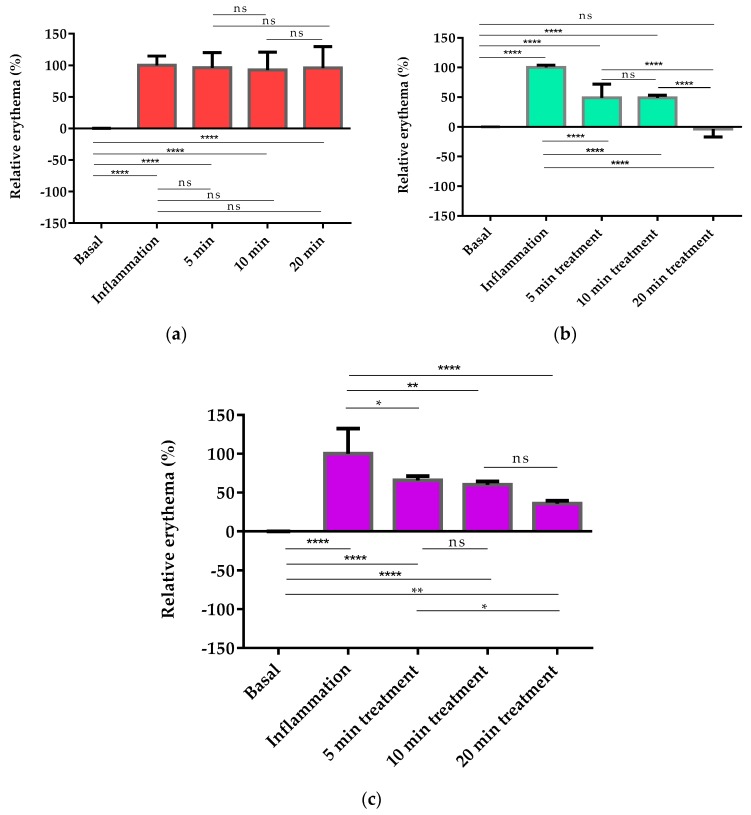
Colorimetric studies for pharmacological evaluation. (**a**) Statistical differences of positive control with respect to basal values (0 min); (**b**) relative erythema (%) of PGZ-limonene at different time intervals with respect to the basal stage (0 min); (**c**) relative erythema (%) of limonene at different time intervals with respect to the basal stage (0 min). Horizontal bars represent the average value. Significant statistical differences: * *p* < 0.05, ** *p* < 0.01, **** *p* < 0.0001, ns = non-significant.

The pharmacological efficacy of PGZ-limonene was evaluated by skin color differences from the backs of mice with respect to basal color. The evolution of erythema can be seen in Figure 3 and Figure S6, which displays the reproduction of the color codes as a sequence through different steps: basal color, induction of inflammation/vasodilation, and treatment after 5, 10, and 20 min. The results showed significant differences between the relative erythema (%) of the topical treatment with PGZ-limonene and limonene with respect to positive control. PGZ combined with limonene reduced the relative erythema below the basal value at 20 min (Figure 4a–c).

#### 2.4.2. Histological Analysis

**Figure 5:**
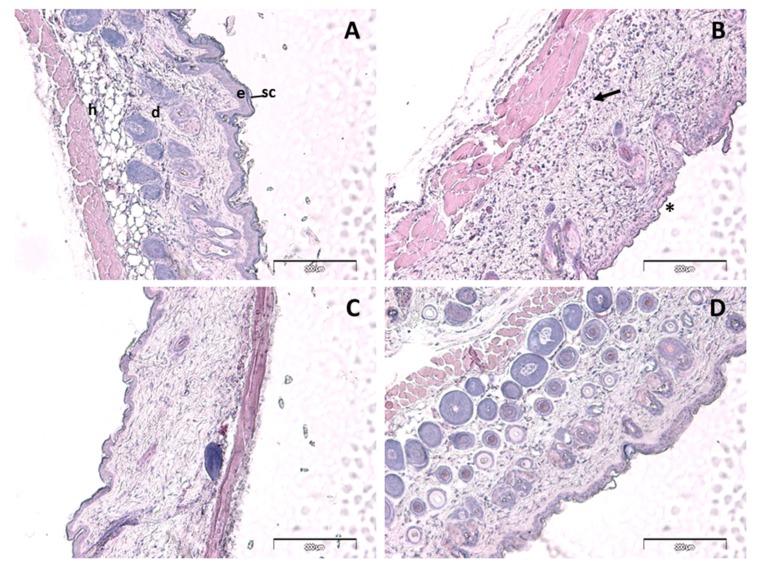
Hematoxylin and eosin staining of control (**A**), *m*-Xylene (**B**), PGZ-limonene (**C**), and limonene (**D**) mice’s back skin from affected area (×10 magnification). Hematoxylin stains nuclei blue/black, and eosin stains keratin and cytoplasm red/orange. Bars = 200 µm. sc = stratum corneum, e = epidermis, d = dermis, h = subcutaneous layer Arrow = leucocyte infiltrate, * = loss of stratum corneum.

Histologically, control skin consisted of a relatively thin epidermis with a contiguous stratum corneum and normal dermal appendages (Figure 5A). A similar pattern of staining was observed in the PGZ-limonene treated skin (Figure 5D). Loss of the stratum corneum was evident in the *m*-Xylene treated mice (*, Figure 5B), along with a prominent leukocyte infiltrate (arrow, Figure 5B) accompanied by a general loss of dermal appendages, including sebaceous glands and hair follicles. PGZ-limonene skin was limited to less infiltrating leukocytes compared to the *m*-Xylene skin and a normal epidermis with a contiguous stratum corneum (Figure 5C).

## 3. Discussion

Rosacea is an inflammatory skin disease which remains incurable because the current treatment has limitations and a significant number of patients are unresponsive to it or have unsatisfactory results [28]. Mounting evidence suggests that PPAR-γ activation is a promising target to regulate pro-inflammatory cytokines expression [38]. Therefore, PPAR-γ agonists such as PGZ could promote an anti-inflammatory effect to treat several diseases [39]. In the present study, the possible application of PGZ in rosacea treatment was evaluated. Validation of the analytical method was carried out in accordance with international conference on harmonization (ICH) guidelines, for which the following criteria were analyzed: linearity, precision, accuracy, robustness, specificity, limits of detection, and quantification. The objective of validating an analytical method is to confirm that the analytical procedure employed for a specific test is suitable for its intended use [40,41]. Spectra showed maximum absorbance at a wavelength of 269 nm and the calibration curve was found to be linear in the concentration range of 1.5–110 μg/mL, with a correlation coefficient (*r*^2^) value of 0.0998 (Figure S1). The obtained values for accuracy did not exceed ±5% (Table S3), and precision was maintained below ±3% (Table S2), thus proving that the analytical method is accurate and precise within the determined concentration range.

Furthermore, the human skin was analyzed as a possible route for PGZ delivery, because topical treatment offers important advantages that include reduced side effects and ease of product use over the target areas [42]. However, poor permeation of the drug through the skin is the primary challenge in the development of topical formulations [43]. The use of penetration enhancers is a common strategy to increase drug flux through the stratum corneum, which is the upper layer of the skin and the major barrier for drug permeation [44,45]. In this study, ex vivo permeation experiments through human skin using PGZ with different penetration enhancers evidenced that terpenes (menthol, cineole, and mainly limonene) were the most effective.

Ex vivo permeation experiments through human skin using PGZ with different penetration enhancers (Figure 1) evidenced that the permeation flow of PGZ without enhancers was relatively low: 0.84 ng/(h/cm^2^). The addition of azone, squalene, linoleic acid, menthol, pyrrolidone, limonene, and cineole changed the permeation flow to 1.94, 0.66, 0.54, 0.38, 0.69, 2.19, and 1.8 ng/(h/cm^2^), respectively. Therefore, limonene was the most effective penetration enhancer that promoted the permeation of PGZ through human skin. As shown in Table 1, limonene presents the highest values for *J_ss_, k_p_, Q_ret_*, and *C_ss_* [44]. Some studies suggest that hydrocarbon terpenes like limonene (log *p* value of 4.53) are more effective to enhance skin penetration of lipophilic drugs like PGZ (log *p* value of 2.3) [45,46]. Terpenes consist of isoprene units that enhance the permeation of hydrophilic and lipophilic drugs [47]. The mechanism of action of limonene as a penetration enhancer is based on changing the structure of lipids between the stratum corneum, with a consequent increase of intercellular diffusivity and improvement of drug partitioning into the tissue [48]. Researchers have found that limonene has anti-inflammatory properties in a murine dermal model, as well as healing effects on the epidermal barrier [49]. However, some researchers have suggested limonene could be toxic for human skin [50,51], whereas others do not consider the research significant enough to invalidate its use [52,53,54]. In addition, topical treatment of normal mouse skin with PPAR does not affect basal transepidermal water loss. In other words, permeability barrier function is not altered [55]. Furthermore, the high retention of PGZ in the human skin (207.65 µg/g of tissue) indicates that limonene promotes the retention of drug in the skin, which could prolong the duration of drug action and increase efficacy in the treatment of rosacea, thus favoring the likelihood of a reduction of the dosing frequency in clinical practice.

The in vitro cytotoxicity studies were made using HaCaT cells after 24 h of incubation with different dilutions of PGZ-limonene and limonene. Figure 2 shows similar results in both cases with cell viability greater than 80% in five of the six dilutions tested. Therefore, PGZ-limonene did not affect cell viability, which suggests the absence of apparent toxicity and suitability for topical use [56,57].

The irritancy test was performed in male albino rabbits. The possibility of causing skin damage is of vital importance in the development of topical treatments [58]. The result after 24 and 72 h of exposure to the formulation at a concentration of 1 mg/mL of PGZ and 5% of limonene was obtained in accordance with previous studies [59,60], showing a primary irritation index below 0.5 in all cases, which indicates that PGZ-limonene and limonene are non-irritant.

Moreover, the relative erythema (%) was determined in order to confirm the ability of PGZ-limonene to reduce erythema and vasodilation, which are the main clinical features of rosacea. Recent studies using animal models of inflammatory skin diseases have confirmed that topical administration of PPAR-γ ligands like PGZ decreases epidermal hyperplasia, enhances permeability barrier function, and reduces the inflammation mediated by T lymphocytes [61]. In this study, a skin colorimetric assay was performed in order to measure the skin color of the mice’s backs after inducing inflammation and treating it with PGZ-limonene and limonene. Topical application of *m*-Xylene immediately leads to vasodilation and erythema. The positive control showed significant differences (*p* < 0.0001) with respect to basal values during the 20 min of assay, though without significant changes over time (Figure 4a). Treatment with PGZ-limonene and limonene (Figure 4b,c) decreased the level of vasodilatation, but not at the same proportion. PGZ associate with limonene significantly reduced the relative erythema in all the time intervals tested (*p* < 0.0001), and at 20 min the differences intensified, decreasing below the basal value with notably reduced erythema, thus resulting in a lighter color. (Figure 3 and Figure 4b). This is likely attributed to limonene because of its drug-enhancing effect [62]. Finally, the application of limonene also significantly decreased redness, reaching a relative erythema of about 40% at 20 min without reaching the basal state, revealing that the benefits of limonene go far beyond its use as a penetration enhancer, because it also has an anti-inflammatory effect. Consequently, its combined use with PGZ constitutes a strategy to increase the pharmacological efficacy of rosacea treatment.

The back skin from mice was used to obtain sections 6 µm in thickness that were stained with hematoxylin and eosin in order to evaluate leukocyte infiltration, as well as histopathological changes after treatment. Control skin exhibited normal morphology (Figure 5A). Dermal papillae created a clearly demarcated border between the epidermis and dermis. The topical application of *m*-Xylene (Figure 5B) caused a loss of the stratum corneum, an absence of epidermal ridges, and dermal papillae, thus resulting in a diminished definition of the border between the epidermis and dermis. Additionally, general loss of dermal appendages such as sweat glands, sebaceous glands, and hair follicles was observed. Moreover, the presence of prominent leukocyte infiltration was evidently manifested as a result of the inflammatory process. In accordance with other studies, the treatment with PGZ-limonene after inducing inflammation notably improved the structural characteristics of the mice skin where a contiguous stratum corneum was observed [63,64]. This treatment significantly attenuated the inflammatory response, which was evident with less leukocyte infiltration compared to the *m*-Xylene skin (Figure 5C). A similar structure to the control was observed in the skin treated with limonene (Figure 5D), which showed normal skin layers and an absence of pathological changes. Therefore, the results of histological and colorimetric studies suggested that PGZ-limonene may be used as a promising therapeutic treatment for rosacea. In summary, the experimental assays carried out in this study indicated the efficacy and the capability of PGZ-limonene to regulate the signaling pathways involved in inflammatory processes.

## 4. Materials and Methods

### 4.1. Materials

The PGZ was purchased from Capot Chemical (Hangzhou, China), and the penetration enhancers and *m*-Xylene were obtained from Sigma-Aldrich (Madrid, Spain). Transcutol was supplied from Gattefossé (Barcelona, Spain). Reagents for histological procedures were purchased from Sigma and Thermo Fisher Scientific (Barcelona, Spain). Reagents for cell culture were obtained from Gibco (Carcavelos, Portugal). The HaCat was acquired from Cell Lines Service (CLS, Eppelheim, Germany) and MTT used for cell viability was obtained from Invitrogen Alfagene^®^ (Carcavelos, Portugal). Water Millipore MilliQ system (Millipore Corporation, Bedford, MA, USA) was used for all the experiments, and all reagents used were of analytical grade.

### 4.2. Validated Analytical Method

To validate the new analytical method, high-performance liquid chromatography (HPLC) was performed. The HPLC system is composed of a Waters 1525 pump and a 2487 UV-Visible detector (Waters, Milford, CT, USA). Data were collected and processed using Empower Pro software (Waters, Milford, CT, USA). The analysis was carried out by a chromatographic column 100 C18 (250 mm × 4.6 mm × 5 μm). The composition of the mobile phase was acetonitrile: ammonium acetate: glacial acetic acid (75:25:2 (*v*/*v*)). The mobile phase was filtered using a membrane filter PVDF of 0.45 μm (Millipore Corp., Madrid, Spain). The mobile phase was pumped through the chromatography column with a flow rate of 0.7 mL/min and 10 μL of injection volume. Detection was performed by UV spectrophotometry at λ of 269 nm.

#### Conditions Analyzed

The standard PGZ stock solution (200 μg/mL) was prepared daily in methanol. The calibration curve was prepared by dilutions in mobile phase in a range of concentration from 1.5 to 110 μg/mL. The method was validated in terms of linearity, precision, accuracy, robustness, sensitivity, and specificity. The validation was carried out according to the ICH Q2A and ICH Q2B.

- Linearity
  The calibration curve was prepared from nine different concentrations of PGZ (1.5 to 110 μg/mL). Three calibration curves were prepared, evaluating the linearity according to the determination coefficient (*r*^2^) of each curve, the y-intercept, the slope of the regression line, and the residual and sum of the squares.
- Precision
  Instrument precision was determined by intermediate precision (inter-day). It was expressed according to the standard deviation (SD) and the % coefficient of variation (CV). The precision was evaluated to analyze sets of three standard samples of 3, 60, and 110 μg/mL within three intercalated days (inter-day). The selected concentrations correspond to the lowest, the intermediate, and the highest concentrations of the calibration curve.
- Accuracy
  The accuracy was determined by measuring the degree of approach between the real value and the experimental data. Accuracy was assessed for concentrations of PGZ (3, 30, 60, and 110 μg/mL) and analyzed in triplicate. The margin of error was calculated for each concentration between the theoretical value ($\chi_{a}$) and the experimental value ($\chi_{r}$) by Equation (1):
  (1) $$\chi_{d}=\frac{\chi_{a}-\chi_{r}}{\chi_{a}}\cdot 100$$
- Robustness
  Robustness was determined by changing experimental flow conditions and the composition of the mobile phase. The flow was varied at ±0.1 mL/min, and the concentration of the mobile phase was varied at ±3% acetonitrile and ±3% ammonium acetate. The effects of these variations on the experimental conditions were tested for retention time. Standard deviation (SD) was calculated.
- Specificity
  The specificity of the method was evaluated by analyzing the possible interferences due to the components of the skin that are released during the passing of the drug with the penetration enhancer. Four different samples were evaluated: blank of mobile phase, standard of 30 ppm, blank of skin as a control, and sample of skin permeated with PGZ-limonene. A volume of 10 μL of each sample was injected, and then the chromatogram profiles (wavelength 269 nm) were analyzed.
- Sensitivity
  Sensitivity was analyzed by the limit of detection (LOD) and the limit of quantification (LOQ). LOD is the lowest concentration of analyte that can be determined, and LOQ is the lowest concentration that can be quantified with adequate accuracy and precision. The signal-to-noise ratio was found by comparing signals from samples of known low concentrations of drug with the signals of blank samples and then establishing the lowest concentration of drug that can be reliably detected, in addition to being reliably quantified. A signal-to-noise ratio of 3:1 for LOD and 10:1 for LOQ were ultimately determined.

### 4.3. Permeation Studies in Human Skin

A healthy 38-year old woman donated a skin sample from her abdominal region and, with written informed consent, facilitated the use of this sample for permeation studies. The consent was obtained in accordance with the Ethical Committee of the Hospital of Barcelona and was assigned the number 001, (dated 20 January 2016). Free-PGZ solution (1 mg/mL), diluted with transcutol/water 4:5.5 (*v*/*v*) and mixed with 5% of different penetration enhancers, were assayed. The penetration enhancers studied were: linoleic acid, squalene, menthol, pyrrolidine, azone, limonene, and cineole (*n* = 3). The study was performed in Franz diffusion cells with diffusion area of 2.54 cm^2^. The experiment was carried out in triplicate using the sample of skin from the same donor to reduce variability due to biological factors. Skin was assessed by measuring transepidermal water loss (TEWL) (TEWL-meter TM210 Courage & Khazaka, Koln, Germany), exhibiting values below 10 g/m^2^·h. Dermatomed skin slices of 0.4 mm thickness were placed between the receptor and donor compartments. Samples of 0.3 mL were placed in the donor compartment and the same volume of samples were extracted from receptor compartment at established time intervals for 31 h and replaced with fresh receptor medium (transcutol/water, 60:40) at 32 ± 0.5 °C under continuous stirring to simulate sink conditions. The quantitative determination of permeated PGZ was analyzed in triplicate by HPLC. Kinetic parameters were estimated using GraphPad Prism^®^ 6.0 (GraphPad Software Inc., San Diego, CA, USA).

### 4.4. Permeation Parameters

The accumulated amounts of PGZ (μg) that were penetrated per cm^2^ of skin were analyzed for the collected samples and plotted against time (h). Permeation profiles were analyzed based on a diffusion model for an infinite dose condition. PGZ flow $(J_{ss},$ μg/(h/cm^2^)) through the skin was calculated by plotting the cumulative amount of drug permeating the skin *versus* time, determining the slope of the linear portion of the curve by analysis of linear regression using GraphPad Prism^®^ 6.0 (GraphPad Software Inc.) and dividing by the diffusion area. The permeability coefficients $(K_{p,}$ cm/h) were obtained by dividing $J_{ss}$ (by the initial concentration of drug ($C_{o}$) in the donor compartment. The steady-state plasma concentration ($C_{ss}$) of drug, which would penetrate the dermal barrier after topical application, was obtained using the following Equation (2):

(2) $$C_{ss}=\frac{J_{ss}\times A}{Clp}$$

where $C_{ss}$ is the steady-state plasma concentration, $J_{ss}$ is the flow, *A* is the area of application, and Clp is the plasma clearance. The calculations are based on a maximum area of application of 1 cm^2^ and human Clp value of 2.26 L/h ± 1.22 [65], in order to ensure the local action of the formulation.

When the permeation study finished, the skin was removed from the Franz cells, cleaned with distilled water, and dried with filter paper. These samples of skin were used to determine the amount of PGZ retained.

The permeated area of the skin was cut and weighed. The PGZ retained in these skin fragments was extracted with 2 mL of methanol after 20 min in an ultrasonic processor. The resulting solutions were analyzed by HPLC, determining the amount of PGZ retained in the skin *Q_ret_* (µg/g skin/cm^2^).

### 4.5. Toxicity in HaCat Cell Line and Skin Tolerance

The effect of PGZ on cell viability was evaluated using the MTT cytotoxicity assay (reduction of tetrazolium salt carried out by intracellular dehydrogenases of viable living cells). To develop this assay, immortalized human keratinocytes (HaCaT) cell line (2 × 10^5^ cells/mL) were plated in 96-wells plates (Corning) and cultured in a humidified incubator at 37 °C in a 5% CO_2_ atmosphere for 24 h to allow adhesion. Experiments were performed at 80–90% of confluence (passes between *n* = 85–95). Cells were grown in high-glucose Dubelcco’s Modified Eagle’s medium (DMEM) supplemented with 25 mM hepes, 1% non-essential amino acids, 100 U/mL penicillin, 100 μg/mL streptomycin, and 10% heat inactivated foetal calf serum (FCS). HaCaT were treated with different concentrations from 0.5 to 0.01 mg/mL of PGZ-limonene and limonene for 24 h and then incubated with fresh medium in presence of 10% MTT (5 mg/mL in phosphate buffered saline) for 2 h at 37 °C. After this, the medium was removed carefully and 100 μL of DMSO 99% purity was added to lysate the cells and the purple insoluble crystals of MTT were dissolved. The cell lysate was transferred to a 96-well new plate and then the absorbance was read using a Microplate Autoreader at excitation/emission of 540/630 nm (Modulus Microplate Multicode Reader-Turner Biosystems, Sunnyvale, CA, USA). In parallel, a negative control (cells without any stimulation or treatment) was processed for comparison. Absorbance values were considered directly proportional to cell viability, and the percentage cell viability was calculated by Equation (3).

(3) $$Cell\;viability=\left[\frac{A\;\mathrm{sample}}{A\;\mathrm{control}}\right]\times 100$$

The potential of skin irritation by PGZ-limonene was assessed by the Draize skin irritation test on New Zealand albino male rabbits (2 kg), which were purchased from San Bernardo farm (Navarra). This test was performed according to the Ethical Committee for Animal Experimentation of University of Barcelona (UB) and followed the respective guidelines [66]. The rabbits were acclimatized for 7 days before the study, after the dorsal area of the trunk was shaved with clippers 24 h before the beginning of the assay. Three groups of animals were analyzed (*n* = 3/group): Group 1:0.9% (*w*/*v*) NaCl solution (Control); Group 2: PGZ-limonene; Group 3: limonene. Two squares were drawn on each side of the back of each rabbit, and a volume of 0.5 mL of each solution was applied on the hair-free skin on each square. This area was covered with gauze and polyethylene film (parafilm^®^) and secured with hypoallergenic sticking plaster. The formation of edema and erythema were analyzed after 24 h and 72 h of exposure. The edema and erythema scores were established according to the degree of severity (graded 0–4). The primary irritation index value was calculated, and the mean value was registered. The treatment was classified according the reported specifications: “non-irritant” (<0.5), “irritant” (2–5), or “highly irritant” (5–8) [66]. The Draize test allowed us to estimate the skin irritation potential but it is not a predictor for skin sensitization potential.

### 4.6. Efficacy Studies

An in vivo model was performed in order to evaluate the efficacy of PGZ for rosacea treatment using the BALB/c backs of mice (four months old). The study protocol was approved by the Animal Experimentation Ethics Committee of the UB with date 28/01/2016 (CEEA/UB ref. 4/16 and Generalitat ref. 8756). Three groups of mice (*n* = 5), including the control group, were assayed. The groups were: positive control (*m*-Xylene), PGZ-limonene, and limonene. The skin color of the backs of the mice was determined using a MPA 5 Multi Probe adapter from Courage + Khazaka electronic GmbH, equipped with a CL400, (Cologne, Germany) The device emits a white LED light that illuminates a circular part of the skin homogeneously. The light scattered by the skin is detected by the colorimeter probe and is expressed as the intensity of light in terms of the three basic light components, R, G, and B (red, green, and blue), on a scale of 0 to 255 each. The skin colorimeter was measured before and after induced vasodilation and erythema by applying *m*-Xylene on the backs of the mice with the help of a sterile gauze. Next, 400 µL of each formulation was promptly applied and PGZ was used at drug concentration of 1 mg/mL and limonene at 5%. Skin color determinations were performed after 5, 10, and 20 min of treatment. Colors were reproduced using Microsoft Excel^®^ software (Version 2016, Microsoft Corporation, Redmond, WA, USA) from the RGB codes and plotted as a sequence and evaluated in accordance with previously mentioned equations [67].

The difference values were calculated between each measurement and the average of basal values. The corrected difference obtained between the basal color and the one after inducing vasodilation (vasodilation difference) was considered 100% erythema. Relative erythema (%) values were calculated by dividing each corrected difference by the vasodilation difference and were plotted as a sequence of the different stages in order to see the evolution of erythema. One-way Analysis of Variance (ANOVA), along with Tukey’s Multiple Comparison Tests, were performed for assessing the statistical significance of both the evolution of erythema and the comparison between treatment and basal values. Statistical analysis was performed using GraphPad Prism^®^ software version 6.0.

### 4.7. Histological Analysis

For histological observation of skin structure, the animals were sacrificed immediately after colorimeter assay by cervical dislocation, and then the skin of the backs of the mice was carefully collected and set overnight in 4% buffered formaldehyde at room temperature. Their back skin was then embedded in paraffin, cut into 6 µm sections, stained with hematoxylin and eosin, and then viewed under a microscope for the evaluation of skin structure and possible inflammatory responses.

### 4.8. Statistics

All the values are expressed as mean ± standard deviation. Statistical analysis was performed using GraphPad Prism^®^ software version 6.0 (GraphPad Software Inc.).

## 5. Conclusions

In summary, our study suggest that PGZ-limonene could be used as a therapeutic treatment for rosacea by improving the underlying inflammatory processes. However, further studies are needed to determine the underlying mechanisms for the anti-inflammatory effect of the drug in addition to providing greater assurance of its safety prior to use in clinical practice.

## Figures and Tables

**Table 1:** Permeation and prediction parameters of PGZ and penetration enhancers.

Permeation and Prediction Parameters	Free-PGZ	Azona	Squaleno	Linoleic Acid	Menthol	Pyrrolidone	Limonene	Cineol
*J_ss_* (µg/(h/cm^2^)) × 10^4^	8.42 ^a,c,d,f,g^	19.40 ^b,c,d,e^	6.56 ^f,g^	5.38 ^f,g^	3.83 ^e,f,g^	6.89 ^f,g^	21.90 ^g^	18.00
	(7.68–9.36)	(17.4–23.3)	(5.81–7.22)	(4.74–6.02)	(3.44–4.31)	(6.30–7.58)	(19.7–25.1)	(15.2–20.8)
*K_p_* (cm/h) × 10^5^	4.92 ^a,c,d,f,g^	12.10 ^b,c,d,e^	3.62 ^f,g^	3.21 ^f,g^	2.33 ^e,f,g^	4.21 ^f,g^	13.20 ^g^	2.53
	(4.33–5.41)	(11.90–14.30)	(3.16–3.98)	(2.69–3.53)	(2.20–2.66)	(3.69–4.53)	(12.90–16.50)	(2.38–2.78)
*Q_ret_* (µg/g skin/cm^2^)	42.61 ^a,c,d,e,f,g^ (38.34–46.86)	8.42 ^b,d,f,g^ (7.67–9.36)	53.61 ^c,d,e,f,g^ (47.24–57.97)	14.84 ^d,f,g^ (14.35–16.32)	101.82 ^e,f^ (90.63–112.00)	18.04 ^f,g^ (15.23–20.84)	207.65 ^g^ (186.88–229.41)	94.74 (85.26–105.21)
*C_ss_* (ng/mL) × 10^4^	3.73 ^a,c,d,f,g^	8.57 ^b,c,d,e,g^	2.90 ^f,g^	2.38 ^f,g^	1.69 ^e,f,g^	3.05 ^f,g^	9.68	9.60
	(3.45–4.20)	(7.71–9.52)	(2.41–3.29)	(2.04–2.62)	(1.44–1.96)	(2.84–3.35)	(8.81–10.50)	(8.54–11.60)

^a^ = Azona; ^b^ = Squaleno; ^c^ = Linoleic acid; ^d^ = Methol; ^e^ = Pyrrolidone; ^f^ = Limonene; ^g^ = Cineol. No differences found for Free-PGZ. Results are expressed by median and range of three replicates. One-way Analysis of Variance (ANOVA) with Tukey’s Multiple Comparison Tests were performed to assess the statistical significance between groups at (*p* < 0.05).

## Supplementary Materials

Supplementary materials can be found at www.mdpi.com/1422-0067/18/12/2548/s1.

## Abbreviations

PGZ	Pioglitazone
PPARs	Peroxisome proliferator-activated receptors
HPLC	Liquid chromatography of high resolution
TLR	Toll-like receptors
IL-8	Interleukin 8
IL-1β	Interleukin 1 β
TNF-α	Tumor necrosis factor α
LOD	Detection limit
LOQ	limit of quantification
*J_ss_*	Permeability flow
*k_p_*	Permeability coefficient
*Q_ret_*	Retained drug
*C_ss_*	Steady-state plasma concentration
GRAS	Generally regarded as safe
ICH	International conference on harmonization
