# Supplementary material for: Human Skin Permeation Studies with PPARγ Agonist to Improve Its Permeability and Efficacy in Inflammatory Processes
Source: Int J Mol Sci. 2017 Nov 28;18(12):2548. doi: 10.3390/ijms18122548 (PMC5751151; doi:10.3390/ijms18122548)
Supplement: Supplementary file 1 [file ijms-18-02548-s001.pdf]

Supplementary Materials

Table S1. Standards of PGZ to analyze the linearity

Standards (μg/mL)	Area 1 (μV*sec)	Area 2 (μV*sec)	Area 3 (μV*sec)	A(Average) (μV*sec)	SD
**1.5**	34167	31506	32387	32387	1355.57
**3**	48311	46324	48380	48311	1167.62
**5**	65374	64878	63891	64878	754.92
**10**	124741	116434	119389	119389	4210.74
**15**	189224	171933	170119	171933	10545.69
**30**	366468	314375	329348	329348	26819.66
**60**	702444	665092	678080	678080	18962.52
**80**	972119	921532	943514	943514	25365.65
**110**	1292669	1309338	1314170	1309338	11280.49

SD: Standard deviation

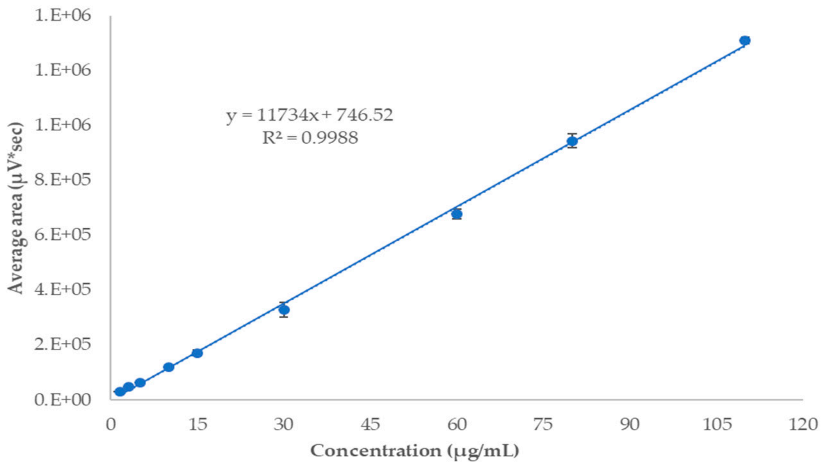

Figure S1: Linearity of the average of 3 calibrate curve. Runs test showed that deviation from linearity is not significant (p=0.1071)

Table S2. Precision inter-day

Day 1		Day 2		Day 3				
Area (μV*Sec)	C (μg/mL)	Area (μV*Sec)	C (μg/mL)	Area (μV*Sec)	C (μg/mL)	SD	CV (%)	Method Precision (%)
45104	3	42679	3	43376	3	1248.49	2.87	97.15
713066	60	711243	60	702444	60	5679.97	0.79	99.19
1295099	110	1293630	110	1292669	110	1223.81	0.09	99.90

C: Concentration; SD: Standard deviation; CV: Coefficient of variation

Table S3. Accuracy of the analytical method

Theor. C. (μg/mL)	Tested C. (μg/mL)	Theor. C. (μg/mL)	Tested C. (μg/mL)	Theor. C. (μg/mL)	Tested C. (μg/mL)	Average (μg/mL)	SD	CV (%)	Relative error (%)	Accuracy (%)
3	3.263	3	3.057	3	3.116	3.145	106.086	3.40	-4.83	104.83
30	30.088	30	29.957	30	30.178	30.074	111.132	0.36	-0.24	100.24
60	59.897	60	59.742	60	57.442	59.027	1374.83	2.30	1.62	98.38
110	109.245	110	109.121	110	110.656	109.674	852.693	0.78	0.29	99.71

SD: Standard deviation; CV: Coefficient of variation

Table S4. Robustness of the analytical method: Effect of the change of flow (mL/min) and variations in the concentration (V/V) of the mobile phase to determine robustness.

Flux (mL/min)	C (μg/mL)	Average retention time (min) ± SD	C (v/v)	Average retention time (min) ± SD
0.6	30<br>60	6.2 ± 0.002<br>6.2 ± 0.007	A:78<br>B:22	5.1 ± 0.002<br>5.1 ± 0.002
0.7	30<br>60	5.3 ± 0.004<br>5.4 ± 0.016		
0.8	30<br>60	4.6 ± 0.008<br>4.5 ± 0.016	A:72<br>B:22	6.1 ± 0.002<br>6.1 ± 0.002

SD: Standard deviation

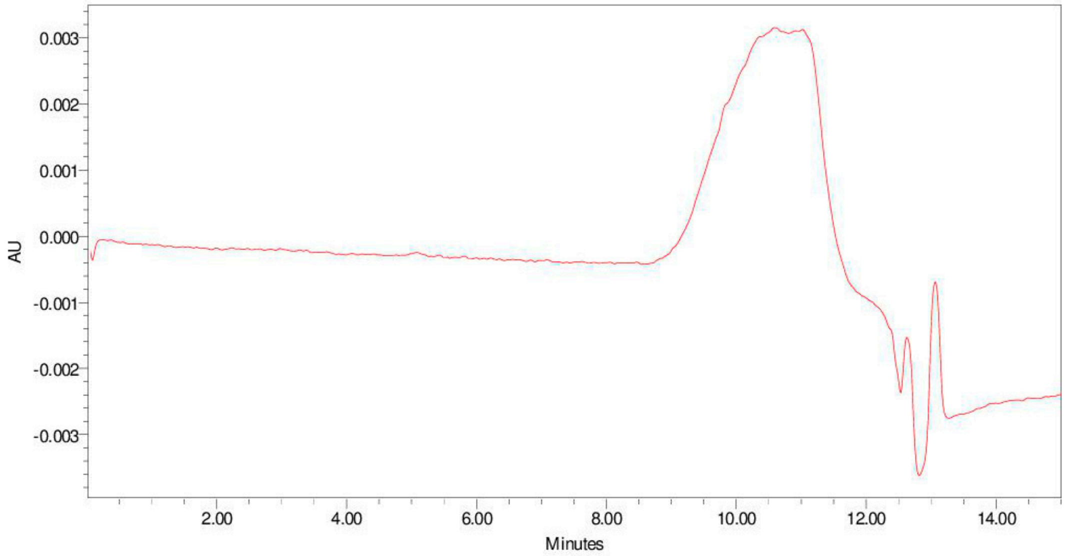

Peak Results

	SampleName	Name	RT	Height	Amount	Units	Area (µV*sec)
1	Blank_control	Pioglitazone	5.390		Missing		

Figure S2: Chromatogram of blank control sample (Mobile phase)

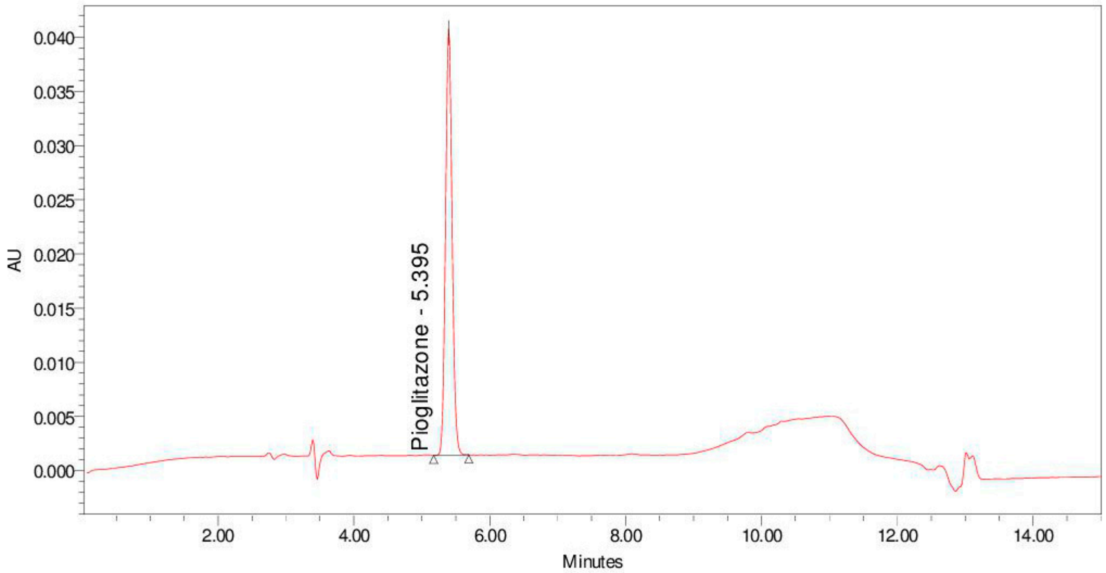

Peak Results

	SampleName	Name	RT	Height	Amount	Units	Area (µV*sec)
1	30 ppm	Pioglitazone	5.395	39301	30.000	ppm	268716

Figure S3: Standard sample 30 ppm.

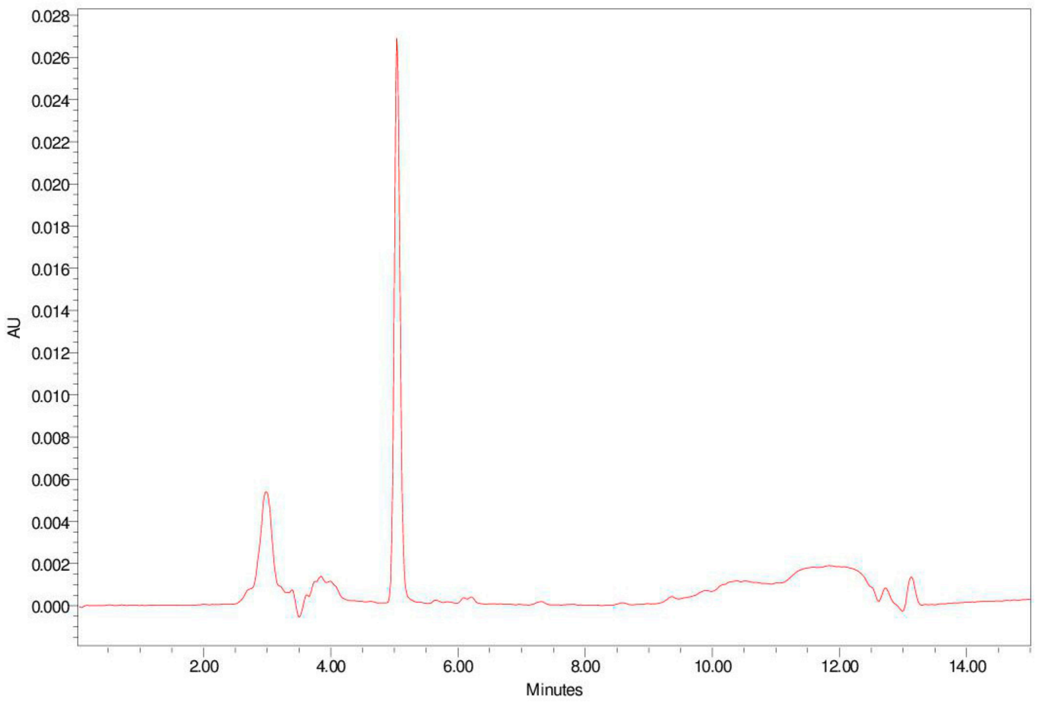

Peak Results

	SampleName	Name	RT	Height	Amount	Units	Area (µV*sec)
1	Time_0_skin	Pioglitazone	5.390		Missing		

Figure S4: Skin blank sample of permeation study as control.

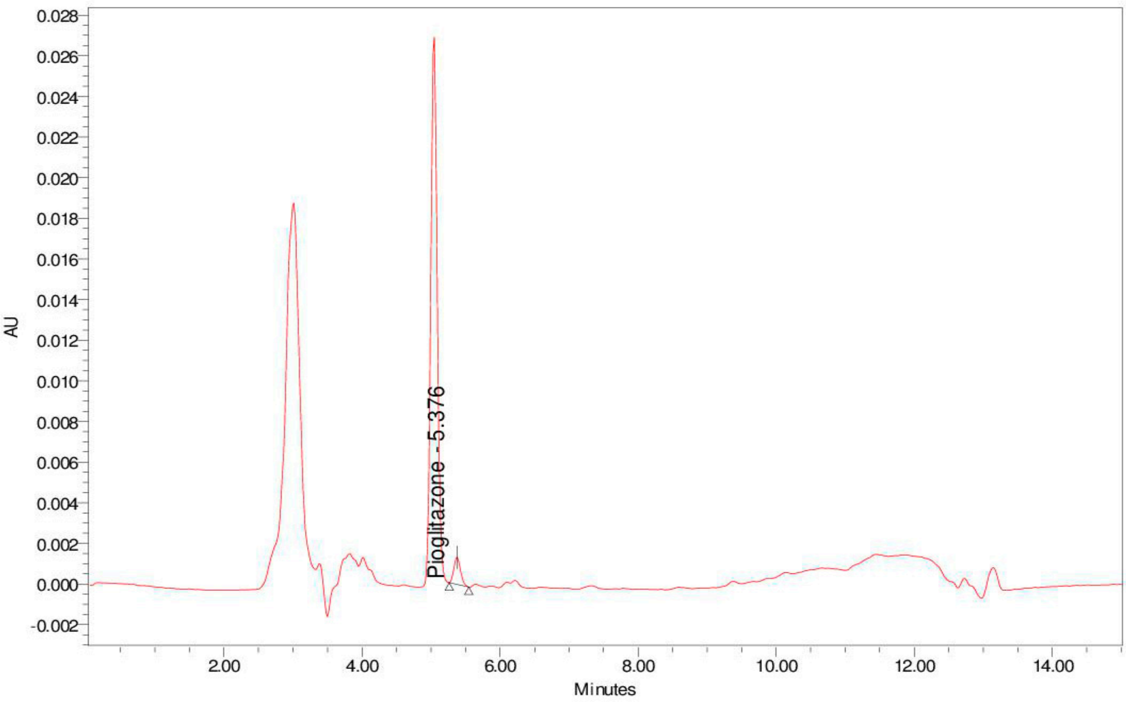

Peak Results

	SampleName	Name	RT	Height	Amount	Units	Area (µV*sec)
1	Limonene_skin_2	Pioglitazone	5.376	1335	0.088	ppm	8967

Figure S5: Skin sample permeation study of PGZ-limonene.

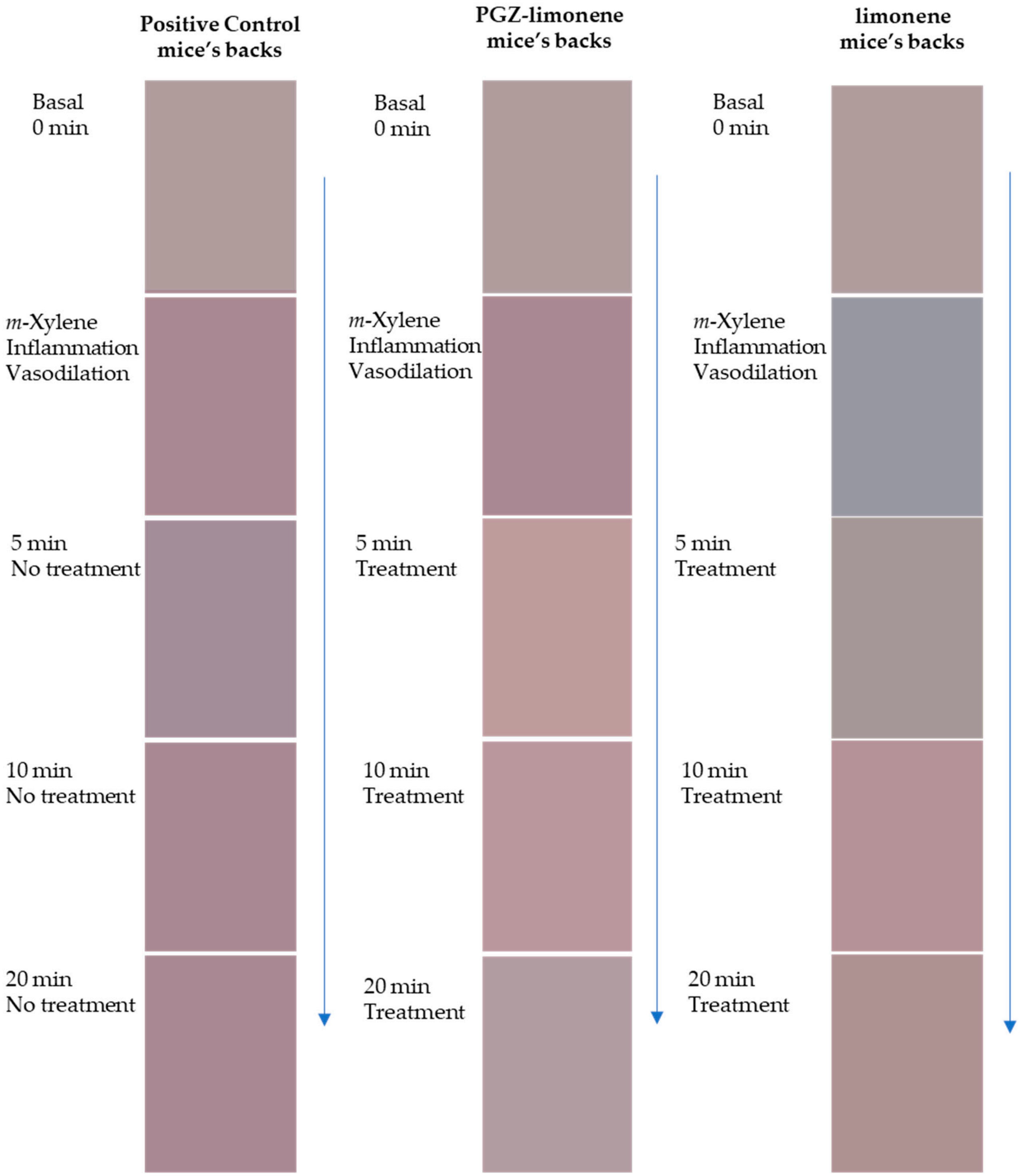

Figure S6.: Evolution of erythema shown as skin color sequence, using PGZ-limonene, limonene, compared with positive control. Colors are reproduced from the average values of RGB codes.
